# Supplementary material for: What Predicts Adherence to Governmental COVID-19 Measures among Danish Students?
Source: Int J Environ Res Public Health. 2021 Feb 13;18(4):1822. doi: 10.3390/ijerph18041822 (PMC7918694; doi:10.3390/ijerph18041822)
Supplement: Supplementary file 1 [file ijerph-18-01822-s001.pdf]

Supplementary Table S1: Questions and answers used in the present analysis derived from the questionnaire of the international student wellbeing survey. For further information please read [16].

**Question**	**Answer categories**
Your gender is	Male; Female; Other
Your age is	[given in years]
Are you currently in a steady relationship?	no, I am single; Yes; It is complicated
Are you born in Denmark	Yes; No
What is the highest level of education your mother has completed?	Less than secondary education; Secondary education (high school); Higher education; Do not know
Indicate which of the following best describes your field of study?	Country specific categorization was used
In which study program are you currently enrolled?	Bachelor program; Master program; Doctoral program; Other, please specify
At which higher-education institution are you currently enrolled?	Country specific list was used
To what extent do you agree with the following statement?<br>'I had sufficient financial resources to cover my monthly costs'<br>Before the COVID outbreak:	Strongly agree; Agree; Neither agree nor disagree; Disagree; Strongly disagree
Where did you mainly live (excluding weekends and holidays)?<br>Before the COVID outbreak:	I stayed with my parent(s); I stayed in a student hall of the university/college; a boarding home or a dormitory; I rented an accommodation with others; I rented an accommodation by myself; Other
On average, how often did you smoke tobacco (cigarettes or cigars)?<br>Before the COVID outbreak:	(Almost) never; Less than once a week; Once a week; More than once a week; (Almost) daily; Prefer not to say
On average, how many glasses of alcohol did you drink in one week?<br>Before the COVID outbreak:	[number of glasses]
On average, how often did you perform vigorous physical activities like lifting heavy things, running, aerobics, or fast cycling for at least 30 minutes?<br>Before the COVID outbreak:	(Almost) never; Less than once a week; Once a week; More than once a week; (Almost) daily
Do you have any of the following underlying conditions?<br>*(multiple options are possible)*	Heart disease; Lung disease (including moderate or severe asthma); Kidney disease; Diabetes; High blood pressure; Immunocompromised conditions (e.g., bone marrow or organ transplantation, HIV, prolonged use of corticosteroids); A recent cancer diagnosis; Obesity; None of these conditions; Prefer not to say
Did you have COVID-19, or do you currently have it?	Yes, confirmed by a lab test; Yes, a health care provider told me that I probably had/have it, but a lab test did not confirm it; I think I had or currently have COVID-19, but a health care provider did not confirm it; No, I do not think I had or currently have it
How worried are you ...<br>... to get infected with COVID-19?	“Not worried at all” until “Very worried” in a score from 0 to 10
Do you know anyone on your personal network that was or currently is infected with COVID-19?	Yes or No

Question	Answer categories
To what degree do you adhere to the COVID-19 measures that are currently implemented by the government?	“Totally not” to “Very strictly” in a score from 0 to 10
Please indicate how much of the time during the past week…<br>…you felt depressed (a)<br>…you felt that everything you did was an effort (b)<br>...your sleep was restless (c)<br>...you were happy (d)<br>...you felt lonely (e)<br>...you enjoyed life (f)<br>...you felt sad (g)<br>...you could not get going (h)<br>…you were bored (i)<br>…you were frustrated with things in general (j)<br>…you felt anxious (k)<br>…you felt calm and peaceful (l)<br>...you lacked companionship (m)<br>...felt isolated from others (n)	None or almost none of the time; Some of the time; Most of the time; All or almost all of the time<br><br>Depression scale – CES-D (Radloff et al. 1991)
Please indicate which of the following statements are true or false:<br>(a) The virus survives for days outside the body in the open air<br>(b) The virus survives for a week outside the body on a plastic surface.<br>(c) Most people who get COVID-19 get very ill.<br>(d) A possible vaccine will take around 12 to 18 months to produce.<br>(e) Smokers who get COVID-19 are more likely to get ill than non-smokers.<br>(f) You can have the virus without any symptoms.<br>(g) On average, children get less ill from the virus than adults.<br>(h) Only elderly people die from COVID-19.	True; False; Don’t know
The questions in this scale ask you about your feelings and thoughts during the last month. In each case, please indicate with a check how often you felt or thought a certain way.<br>1. How often have you felt that you were unable to control the important things in your life?<br>2. How often have you felt confident about your ability to handle your personal problems?<br>3. How often have you felt that things were going your way?<br>4. How often have you felt difficulties were piling up so high that you could not overcome them?	Never; Almost never; sometimes; Fairly often; Very often<br><br>Short form of Cohen’s perceived stress scale (Cohen et al. 1983)
